# The plant hormone auxin directs timing of xylem development by inhibition of secondary cell wall deposition through repression of secondary wall NAC‐domain transcription factors

**DOI:** 10.1111/ppl.12766

**Published:** 2018-08-02

**Authors:** Christoffer Johnsson, Xu Jin, Weiya Xue, Carole Dubreuil, Lina Lezhneva, Urs Fischer

**Affiliations:** ^1^ Umeå Plant Science Centre, Department of Forest Genetics and Plant Physiology Swedish University of Agricultural Sciences Umeå Sweden; ^2^ Stora Enso AB Falun Sweden

## Abstract

Wood formation in higher plants is a complex and costly developmental process regulated by a complex network of transcription factors, short peptide signals and hormones. Correct spatiotemporal initiation of differentiation and downstream developmental stages is vital for proper wood formation. Members of the NAC (NAM, ATAF1/2 and CUC) family of transcription factors are described as top level regulators of xylem cell fate and secondary cell wall (SCW) deposition, but the signals initiating their transcription have yet to be elucidated. We found that treatment of *Populus* stems with auxin repressed transcription of *NAC* transcription factors associated with fiber and SCW formation and induced vessel‐specific NACs, whereas gibberellic acid (GA) induced the expression of both classes of NAC domain transcription factors involved in wood formation. These transcriptional changes were reflected in alterations of stem anatomy, i.e. auxin treatment reduced cell wall thickness, whereas GA had a promotive effect on SCW deposition and on the rate of wood formation. Similar changes were observed on treatment of *Arabidopsis thaliana* stems with GA or the synthetic auxin NAA. We also observed corresponding changes in *PIN5* overexpressing lines, where interference with auxin transport leads to premature SCW deposition and formation of additional fiber bundles. Together, this suggests wood formation is regulated by an integrated readout of both auxin and GA, which, in turn, controls expression of fiber and vessel specific NACs.

**Abbreviations**
CESA: cellulose synthase A
CLV: clavata
CZ: cambial zone
GA: gibberellic acid
GUS: β‐glucuronidase
KNAT1: knotted‐like from *Arabidopsis thaliana* 1
KNOX: knotted‐like homebox protein
NAC: NAM ATAF1/2 and CUC domain containing protein
NST1: NAC secondary wall‐thickening promoting factor 1
OE: over expression/expressor
PCD: programmed cell death
PCW: primary cell wall
PXY: phloem intercalated with xylem
SAM: shoot apical meristem
SCW: secondary cell wall
SND1: secondary wall‐thickening promoting 1
STM: shoot meristemless
SWN: secondary wall NAC
TF: transcription factor
TZ: transition zone
VND: vascular‐related NAC‐domain
WOX: WUSCHEL‐related homeobox

## Introduction

As plants adapted to life on land, some 470 million years ago, the evolution of reinforced vascular tissues conferred an advantage as it allowed individuals to outgrow neighbors competing for sunlight. As this arms race continued, taller plants required yet more structural support, an adaptation that took the form of wood. This secondary tissue allowed plants to withstand severe environmental challenges, such as winds and heavy snow, while at the same time facilitating transport of nutrients from the roots and photosynthates from aerial tissues to sink tissues in the stem and roots. The bulk of plant biomass is located in the thick secondary cell walls (SCWs) of the cells that make up wood. In angiosperm trees, these cells are mainly fibers, vessel elements and tracheids, while gymnosperm wood is composed primarily of tracheids. The importance of woody tissues is reflected in the functional conservation of the biosynthetic machinery, and its regulatory elements, across evolutionarily diverse lineages. For example, the presence of conserved cis‐elements in *CELLULOSE SYNTHASE* (CESA) promoters from *Arabidopsis, Eucalyptus* and *Populus* (Creux et al. 2008) illustrates that the central parts of the regulatory mechanism have changed little in the 82–127 million years since these species diverged (Clarke et al. 2011). Woody tissues arise from derivatives of stem cells in the vascular cambium (Johnsson and Fischer 2016), the regulation of which is carried out, to a large extent, by the same factors as it governs the maintenance and development of the shoot apical meristem (SAM). These consist of, e.g. short peptides, transcription factors (TFs) and hormones (reviewed by Zhang et al. 2014). Previous reports have made clear that many of these factors, such as *CLAVATA* (*CLV*), *PHLOEM INTERCALATED WITH XYLEM* (*PXY*) and *WUSCHEL‐related HOMEOBOX* (*WOX*) homologs have retained their functions in both meristems (Johnsson and Fischer 2016, Somssich et al. 2016). However, two factors known to be involved in the maintenance of stem cell populations in the SAM, the Class I knotted 1‐like homeobox (KNOX) transcription factors shoot meristemless (STM) and knotted‐like from *Arabidopsis thaliana* 1 (KNAT1) were recently shown to be required for differentiation of cambial stem cell derivatives in secondary growth (Liebsch et al. 2014), signifying that they have acquired a novel function during the evolution of secondary growth, opposite to that in primary growth, where their function is to maintain the stem cell population. Cambial derivatives are gradually transitioned out of the cambial zone as additional cell divisions push the layer of stem cells radially outwards. Once outside the cambial zone, cells acquire a specific fate and subsequent differentiation programs are executed. Vessel cells undergo rapid drastic expansion, after which thick SCWs are deposited and the cell undergoes programmed cell death (PCD). Fiber cell differentiation follows a more gradual process, with expansion and deposition of a thin primary cell wall (PCW) followed by a slow maturation phase during which a thick SCW is deposited through the action of a large set of proteins involved in the synthesis, transport and polymerization of various cell wall components. The expression of the genes encoding proteins active during this biosynthetic program, in turn, is controlled by a complex network of transcription factors, many of which belong to the NAC (for NAM, ATAF1/2 and CUC)‐domain family (for a review of this TF family, see Nuruzzaman et al. 2013).

At the top of this hierarchical network lie a number of early expressed transcription factors, often referred to as master switches, which have been implicated in directing the cell fate of cambial stem cells (Kubo et al. 2005, Zhong et al. 2006), as well as the initiation of the transcriptional program directing SCW formation. The secondary wall‐associated NAC domain 1 (SND1), NAC secondary wall thickening promoting factor1 (NST1) and different vascular‐related NAC‐domain (VND) TFs, known as secondary wall NACs (SWNs), have so far been identified as the main effectors in the top tier of this network. SND1 and NST1 have been described to promote thickening of the SCW in *Arabidopsis* fibers, whereas VND TFs are thought to be specifically expressed in, and direct the fate of, vessel cells (for review see Schuetz et al. 2013). With the relatively recent whole genome duplication (WGD) of the ancestor of Salicaceae (Tuskan et al. 2006), a number of these highly conserved genes are now present as multiple paralogs in *Populus* (Sundell et al. 2017). Such duplications may allow for neo‐functionalization of one of the paralogs, either by mutation of the gene itself or by its promoter. As such development has not been previously described in *Populus* SWNs, there is a need to examine the expression patterns of these genes in *Populus*.

The upstream factors, which control the transcription of these master switches, are largely unknown. It has been speculated that concentration gradients of particular hormones, especially auxin and gibberellic acid (GA), define distinct niches in which the different transcriptional programs required for wood formation are induced (Immanen et al. 2016, Bhalerao and Fischer 2017). A recent publication (Immanen et al. 2016) described the variation of the plant hormones auxin, gibberellin and cytokinin across wood‐forming tissue and shows the presence of distinct concentration maxima for each hormone, identifying distinct peaks for auxin in the cambial zone, at the point where cell division takes place, and gibberellin in the SCW‐forming zone, where fibers mature and deposit their thick SCWs. GA may be transported as inactive GA_12_ through the starch sheath from primary tissues to the site of secondary growth (Johnsson and Fischer 2016). Regulation of transport and activation of GA may allow rigid distribution control of active GA to the developing secondary xylem. A major effect of the presence of active GA is to alleviate transcriptional repression by formation of a GA‐GID1‐DELLA complex, dissociating DELLA proteins from promoter regions and subsequently targeting them for degradation (Ueguchi‐Tanaka et al. 2005). Although the pathway of GA signaling affecting wood development is still poorly understood, it was reported that overexpression of any one of the four *PttGID1* homologs in *Arabidopsis* resulted in phenotypes similar to those of constitutive overexpressors of *GA20OX1*, i.e. increases in xylogenesis, elongation and growth rate, concluding that GA signaling plays an important role in xylogenesis (Mauriat and Moritz 2009).

Gene expression in response to auxin is regulated on the transcriptional level in a manner highly similar to that of GA response. Heterodimerization of auxin response factor (ARF)‐proteins with Aux/IAA proteins results in deregulation of auxin‐responsive genes. In the presence of auxin, Aux/IAA proteins dimerize with the Skp, Cullin F‐Box containing TIR1 SCF^TIR‐1^ complex leading to ubiquitin‐mediated proteosomal degradation of the AUX/IAA repressors. Reduction of Aux/IAA proteins then allows for formation of ARF dimers, which, in turn, can bind to promoters of their target genes (for review see Chapman and Estelle 2009).The auxin‐binding affinities of the co‐receptor vary between combinations of different Aux/IAAs and TIR1 homologs (Calderon Villalobos et al. 2012) implying that the *Aux*/*IAA* gene family may account for a wide range of distinct auxin affinities through the sheer number of possible Aux/IAA‐TIR1 combinations.

Together, GA and auxin may act in concert to direct differentiation of cambial stem‐cell derivatives to fiber cells; specifically, gradual reduction in auxin concentration with increasing distance from the cambium and concurrent increase concentration of active GA may constitute a signal for cells to transition from an expansive phase to a maturation one (Immanen et al. 2016). Recognition of such a signal may require receptors with varying affinities for the respective hormones, which, in the case of auxin, can be mediated through differential expression of the abovementioned Aux/IAAs (Bhalerao and Fischer 2014). Given the observation of distinct, but adjacent, concentration maxima for auxin and GA and the co‐incidence of xylem fiber differentiation in the zone between them, we hypothesize that interplay between these hormones may act as a signal to initiate fiber differentiation.

Although the knowledge of the inner workings of how SCW deposition is regulated has increased greatly in recent years, the question of what initiates the transcriptional program itself still goes unanswered. Previous evidence linking the action of plant hormones to the development of new tissues and plant organs led us to hypothesize that the control of SCW regulation may be initiated in reaction to a hormonal stimulus (Johnsson and Fischer 2016).

Our studies were conducted using two model plant systems: *A. thaliana*, a fast growing, herbaceous plant, and *Populus tremula* × *tremuloides*. Both systems have several advantages, such as their genomes being publicly available (AGI 2000, Tuskan et al. 2006). However, *Arabidopsis'* relatively short rotation allows for fast investigation of gene function and expression domains; while it does develop fibers, it only produces limited amounts of wood‐like tissues in the hypocotyl root (Johnsson and Fischer 2016). *Populus*, on the other hand, provides the means for studying effects on a true tree, meaning that questions relevant to economic interests, such as biomass production, cell wall polymer content ratios and degradability, may be answered. However, one of the main obstacles with using *Populus* is that generating transgenic lines is still rather time consuming. Recent advances in transcriptional profiling (Sundell et al. 2017) of genes in *Populus* with high spatial resolution allowed us to partially bypass this limitation and investigate scientific questions using a correlative‐ or systems‐approach, rather than a functional or purely descriptive methodology.

We identified the *Populus* SWNs by a combination of phylogenetic and spatially highly resolved gene expression analyses. Characterization of cis‐elements in their promoters hinted at hormonal regulation of SWNs, specifically by GA and auxin. We found that the *Populus SND*/*NST* genes were induced by GA treatment and had a tendency to be repressed by auxin, while both hormones could induce tested VNDs. The same result was observed in GA‐treated *Arabidopsis* stems, although the effect of NAA treatment on transcription was inconclusive, likely because of fiber cells constituting a miniscule part of the tissue, meaning changes became undetectable. We performed a hormone supplementation experiment, where effects of hormone application on wood formation in decapitated *Populus* were studied. In line with our expectations from the gene expression study, auxin leads to reduced SCW formation, while early cell differentiation appeared unaffected. Treatment by GA significantly reduced the number of differentiated vessels, and we observed a rescue of the SCW maturation gradient across wood as compared with intact controls. Replication of this experiment in *Arabidopsis* yielded similar results, with cell walls of NAA‐treated plants failing to thicken and lignify. In PIN5 overexpressor plants, where auxin is kept from entering the polar auxin transport chain, cell wall thickness is altered and ectopic fiber bundles form. These results pave the way for further studies into the control of wood formation, specifically the factors governing cell differentiation, but also more applied investigations into the effects of differential sensitivity to auxin across the wood maturation zone.

## Materials and methods

### Co‐expression networks

*Populus* gene co‐expression networks as well as heatmaps and expression profiles were generated using the AspWood tool (http://aspwood.popgenie.org/aspwood‐v3.0/), a resource developed and described by Sundell et al. (2017) and built of RNA sequencing transcriptome data generated from tangential cryo‐section series of four wild‐grown *P. tremula* trees. These section series cover a region from the previous years' annual ring, through the cell death zone, secondary xylem, expansion zone, vascular cambium and secondary phloem.

**Figure 1:**
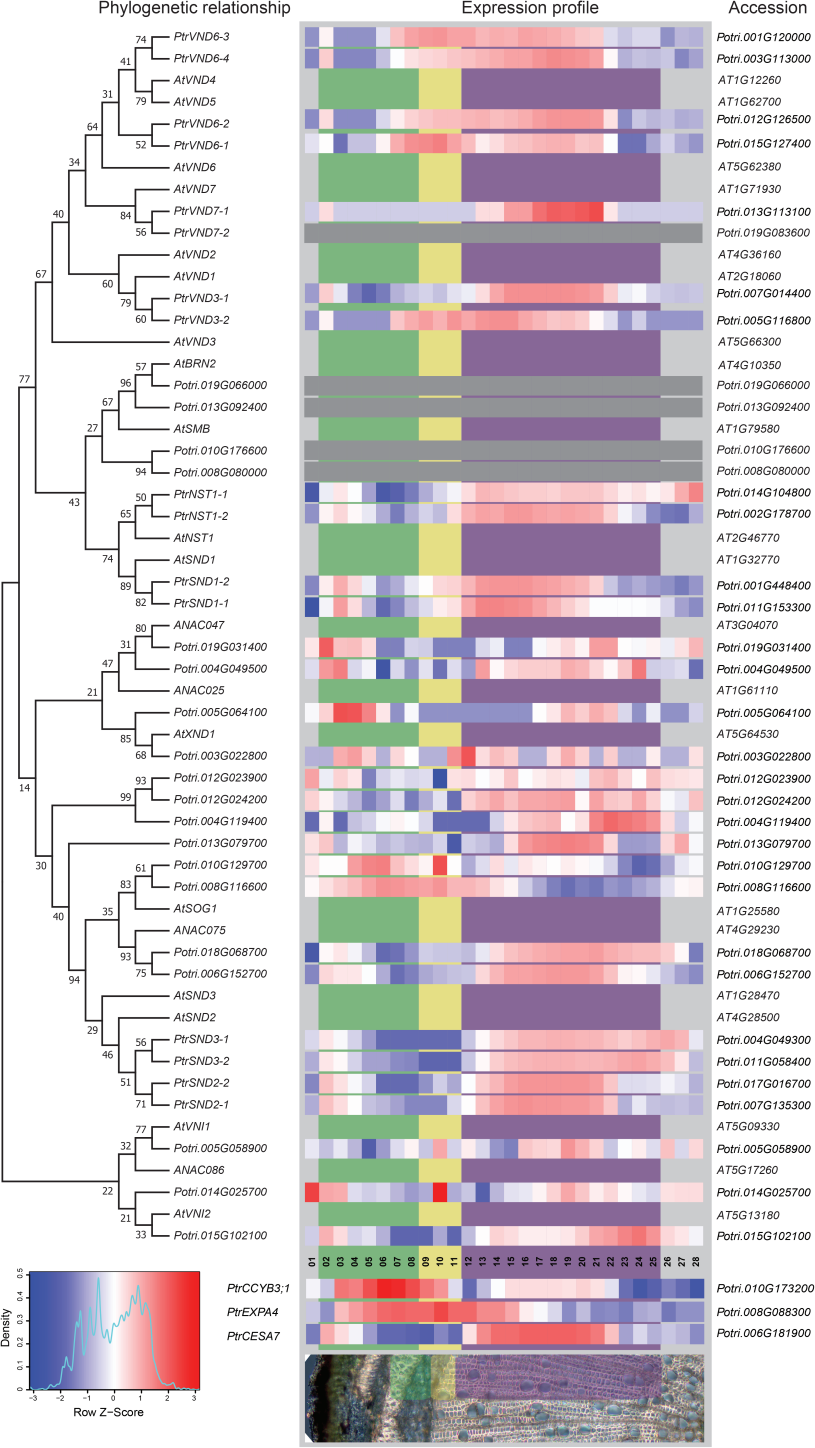
Phylogenetic tree containing *Populus* and *Arabidopsis* fiber and vessel‐related NAC domain transcription factors, as well as expression profiles of these genes across *Populus* wood. Genes of the *VND*‐, *SND1*/*NST1* and *SND2*/*3* families subcluster based on their protein sequence. These differences are also reflected in the expression patterns of their constituent genes in *Populus*. Expression of *VND* genes typically initiates in sections closer to the cambium (green bar), while genes of the *SND1*/*NST1* clade are expressed later (yellow bar), finally transcription of *SND2*/*3* genes is initiated in the SCW maturation zone (purple bar), at the same location as a SCW‐associated *PtrCESA7* (*Potri.006G181900*). Red corresponds to high and blue corresponds to low expression. Expression (VST) is scaled.

**Figure 2:**
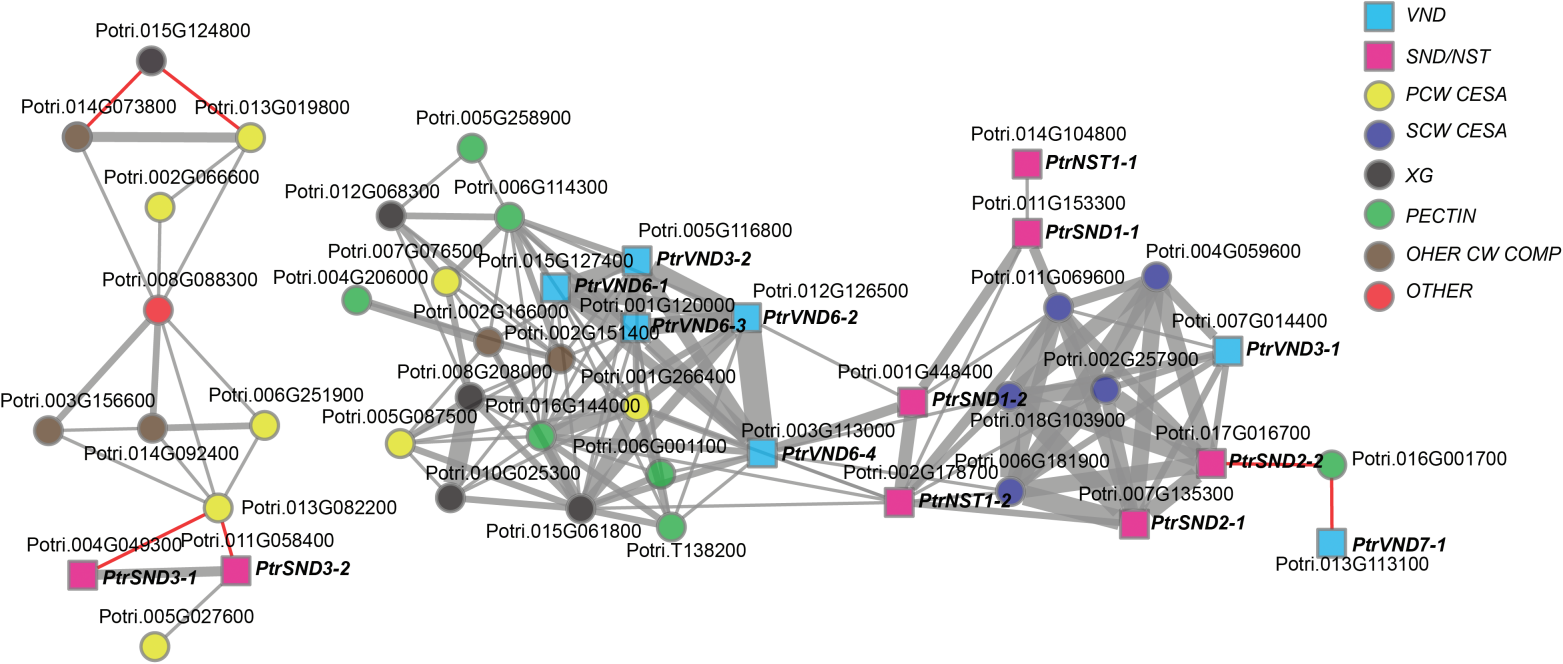
Co‐expression network of *Populus* SWNs, VNDs, as well as PCW and SCW biosynthetic genes. *VND* genes subcluster closely with PCW biosynthetic genes while *SND1*/*NST1* genes occupy a separate cluster, closer to that of the SCW‐associated *CESA*s and *SND2*.

A complete list of genes is available in Appendix S1. To better illustrate differences found in this large network, an un‐biased selection of *CESA*s, as well as cell wall polymer biosynthetic enzymes described earlier (Sundell et al. 2017), along with the SWNs included in the phylogenetic tree of Fig. 1 was used to construct a minimal network (Fig. 2 constituent gene accessions found in Appendix S1).

Genes for construction of the neighbor networks in Fig. 4 were selected to represent each of the gene families: auxin transporters, auxin response factors, GA transporters and response factors, as well as the *SND1*, *NST1* and *VND* orthologs previously identified as being of interest during the construction of the phylogenetic tree and heatmap (see Appendix S1 for accessions). These genes were fed into the AspWood algorithm and a network was constructed where edges of threshold values 4 or higher were displayed.

### Bioinformatics

In silico cloning, construct maps and mapping of cis‐elements to promoter sequences were all performed using CLC Main Workbench (Qiagen Aarhus A/S, Denmark).

The maximum likelihood tree of Fig. 1 was constructed by the mega7software suite (V7.0.26) (Kumar et al. 2016), aligning sequences by MUSCLE (Edgar 2004) and testing phylogeny by the bootstrap method (1000 replications).

### Cloning and transformation

Promoter fragments were amplified from *P. tremula × tremuloides* gDNA using two sets of primers (Appendix S1), resulting in two different fragments (Fig. S3) of the promoter of *SND1* homolog (*SND1‐1*) *Potri.011G153300*. Isolated fragments were cloned into the pCambia1301 vector using restriction enzymes XbaI and HindIII and subsequently applied to transform One ShotTOP10 chemocompetent *Escherichia coli* (ThermoFisher, Göteborg, Sweden) cells for amplification. Amplified plasmids were recovered using a plasmid miniprep kit (Invitrogen, Göteborg, Sweden) and transferred into *Agrobacterium tumefaciens* strain GV3101 PMP90 using electroporation and employed to transform *Arabidopsis thaliana* (Columbia 0 ecotype). The *AtSND1* promoter was amplified with primers including a KpnI and BamHI site (Appendix S1), respectively, and cloned into pCambia1301.

*PttPIN5* was amplified from leaf cDNA using PttPIN5 forward (attB1) and PttPIN5 reverse (attB2) primers (Appendix S1). The amplicon was inserted into pDONR 201 vector using BP‐reaction and afterward transferred into pGKPGWG (Zhong et al. 2008).

### GUS staining

Staining of histological samples to make visible the presence of the *β‐glucuronidase* (GUS) enzyme (as described by Jin et al. (2015)) was performed on internode sections collected from transformed *Arabidopsis* plants. Harvested samples were immediately placed in prechilled acetone for 20 min, after which they were rinsed with chilled water. Subsequently, GUS‐staining solution (Jin et al. 2015) was added to the internodes in 15 ml Falcon tubes (Sarstedt, Nümbrecht, Germany) and the samples were incubated at 37°C for 4 h.

### Quantitative real‐time polymerase chain reaction

Quantitative real‐time polymerase chain reaction (qRT‐PCR) was performed as described in Liebsch et al. 2014, using the LightCycler480 instrument (Roche Molecular Systems, Inc., Rotkreuz, Switzerland) and associated reagents (LightCycler® 480 SYBR Green I Master; Roche Molecular Systems Inc. ). Collected data were processed using the LightCycler 480 Software [release 1.5.0 SP3 (1.5.0.39); Roche Molecular Systems Inc.] and subsequent calculations of relative expression levels made using Microsoft Excel 2010 (Microsoft Corporation, Redmond, Washington).

### Microscopy and image processing

Light microscopy imaging was performed using a Zeiss Axioplan 2 (Carl Zeiss Microscopy, Jena, Germany) and images processed using Adobe Photoshop CS6 (Adobe Systems Incorporated, San Jose, CA). Confocal microscopy was performed on the same sections, stained with Calcofluor white prior to imaging, using a Zeiss LSM780 (Carl Zeiss Microscopy). Staining procedure and confocal settings have been described previously (Liebsch et al. 2014).

### GA/NAA treatment protocol

GA_3_ (Sigma‐Aldrich, Schnelldorf, Germany) and 1‐naphtaleneacetic acid (NAA; Sigma‐Aldrich) were dissolved in separate 1 ml aliquots of lanolin (Sigma‐Aldrich) in 2 ml Eppendorf tubes to 10 and 1 μM concentrations, respectively. Mock aliquots were prepared with an equal volume of ethanol.

The diameter of the 11th internode from the apex of *Populus* (*P. tremula* × *tremuloides*, T89) side shoots was measured and subsequently cut with a scalpel at the 10th internode. For *Arabidopsis*, axillary shoots were removed and the main stem cut immediately below the most basal node. In both experiments, tubes containing lanolin and hormone (with lanolin and ethanol as mock controls) were affixed to the cut end, partially immersing the internode or stem in lanolin. Tubes were left for 7 days before being replaced with new tubes (all prepared at the same time and stored at −20°C). After a total of 3 weeks, samples were harvested and tissues fixed in 4% formaldehyde (ThermoFisher) in phosphate buffered saline (PBS) buffer for 2 h, before being washed three times in ddH_2_O and stored at 4°C in water over night and stained with Calcofluor white, after which they were imaged using a Zeiss Axioplan (Carl Zeiss Microscopy, Jena, Germany).

Short‐term treatments of *Arabidopsis* inflorescence stems and *Populus* internodes were performed by harvesting 10 mm basal pieces of *Populus* or *Arabidopsis* stems and submerging them in liquid half MS medium, supplemented with the same concentrations of GA or NAA used for the long‐term treatment for 4 h.

### Note on accession names

For this work, genetic material was isolated from *P. tremula* x *tremuloides* (*Ptt*) unless stated otherwise, for use in DNA and RNA extractions, and quantification of gene expression used primers designed for this ecotype. Characterization of transcript abundance using the AspWood database utilized sequence annotations from *P. trichocarpa* (*Ptr*) and phylogenetic study of NAC‐TFs (Fig. 1) utilized *Ptr* protein sequence data.

## Results

In an effort to identify candidates for elucidation of phyto‐hormonal control of wood formation, we made use of a previously published, high resolution RNAseq‐database (Sundell et al. 2017), the aligned full protein sequences of all *Populus* NAC transcription factors expressed in wood (see Fig. S1) and their respective *Arabidopsis* homologous. Generating a phylogenetic tree and adding a high spatial RNAseq data for each of the *Populus* NAC in the tree allowed us to identify NAC‐transcription factors expressed in the late cambial and early developing wood zone. In both the phylogenetic tree and the expression profiles, we observe clear separation between *VND*‐family homologous and *SND1* and *NST1* homologous (Fig. 1). While the expression of *VND*‐genes tends to initiate in the cambial zone (Fig.1, green background), with little or no expression in the phloem, *SND1* and *NST1* homologous show moderate expression in the phloem, possibly related to formation of phloem fibers, while major expression initiates in early xylem expansion zone (Fig. 1, yellow background). Homologous of *AtSND2* and *AtSND3* all peak further in to the xylem maturation zone (Fig. 1, purple background), with moderate expression peaks in the phloem similar to those of *SND1*/*NST1* homologous. For comparison, we investigated the expression profile of secondary wall‐associated *CESA* genes and find that the expression profiles of these genes overlap most closely with those of the *SND2* and *SND3* TFs. Additionally, *PtrCYCB3;1* and *PtrEXPA4* (Fig. 1) were found to exhibit expression patterns distinctly different from those of SWNs, with peaks mainly in the cambial zone, in line with their respective roles in cell proliferation and expansion.

While expression patterns within the paralog pairs of *PttSND1, PttNST1*, *PttSND2*, *PttAtSND3* and *PttVND6* are highly similar, we observe divergent expression profiles within the *PttVND3* and *PttVND7* pairs. Expression profiling in the whole plant indicates that both *PttVND7* paralogous are indeed expressed in tissues with primary vasculature (Fig. S4). In line with a function in protoxylem specification, as described earlier (Kubo et al. 2005), the expression of *PttVND7‐2* was not detectable in the secondary xylem (Fig. 1). *PttVND7‐1* was expressed solely toward the end of the maturation zone indicating that this paralog has taken on a novel function.

To verify the differences between these clades, we investigated the overlap between closely neighboring, co‐varying genes *PtrSND1‐1*, *PtrSND2‐2*, *PtrVND6‐3*, *PtrVND7‐1* (Fig. S2B) and found that the neighborhoods of the former two share seven genes, while the neighborhoods of *PtrVND6‐3* and *PtrVND7‐1* have no shared genes. The neighborhood of *PtrVND7‐1* and *PtrSND2‐2* shares six genes although neighborhoods of neither *PtrVND* genes share any genes with *PtrSND1‐1*. In contrast, the paralogs *PtrSND1‐1*, *PtrSND1‐2* have 70 common genes in their neighborhoods. Hence, there is a certain overlap within clades but not between them. Furthermore, different kinds of genes within each neighborhood differs greatly, with *PtrSND1‐1*'s and *PtrVND6‐3's* respective neighborhoods containing TFs known to be involved in wall formation, while those of *PtrSND2‐2* and *PtrVND7‐1* contain a great number of cell wall biosynthetic genes.

Furthermore, the apparent differences between the *PtrSND* and *PtrVND* clades were investigated by generating a co‐expression network composed of *Populus* SWN genes (see Appendix S1 for genes included), *Populus* PCW and SCW *PtrCESA* genes, as well as xyloglucan and pectin biosynthetic genes associated with primary wall formation (Sundell et al. 2017). We observed three distinct subclusters with member genes closely co‐varying. *PtrVND6* orthologs together with *PtrVND3‐2* formed a cluster connected to the bulk of pectin and xyloglucan biosynthetic genes (Fig. 2), as well as three PCW‐associated *PtrCESAs*. *PtrSND1* and *PtrNST1* orthologs were most closely connected to SCW *PtrCESAs*, with the same cluster also including both *PtrSND2* orthologs. The *PtrSND3* orthologs subcluster distinctly from the other SWNs together with PCW *PtrCESAs* and other cell wall biosynthetic genes. While the majority of genes co‐vary positively, *PtrSND3* orthologs are negatively correlated with the rest of the genes in their cluster, apart from a single PCW *PtrCESA* gene (*Potri.005G027600*).

**Figure 3:**
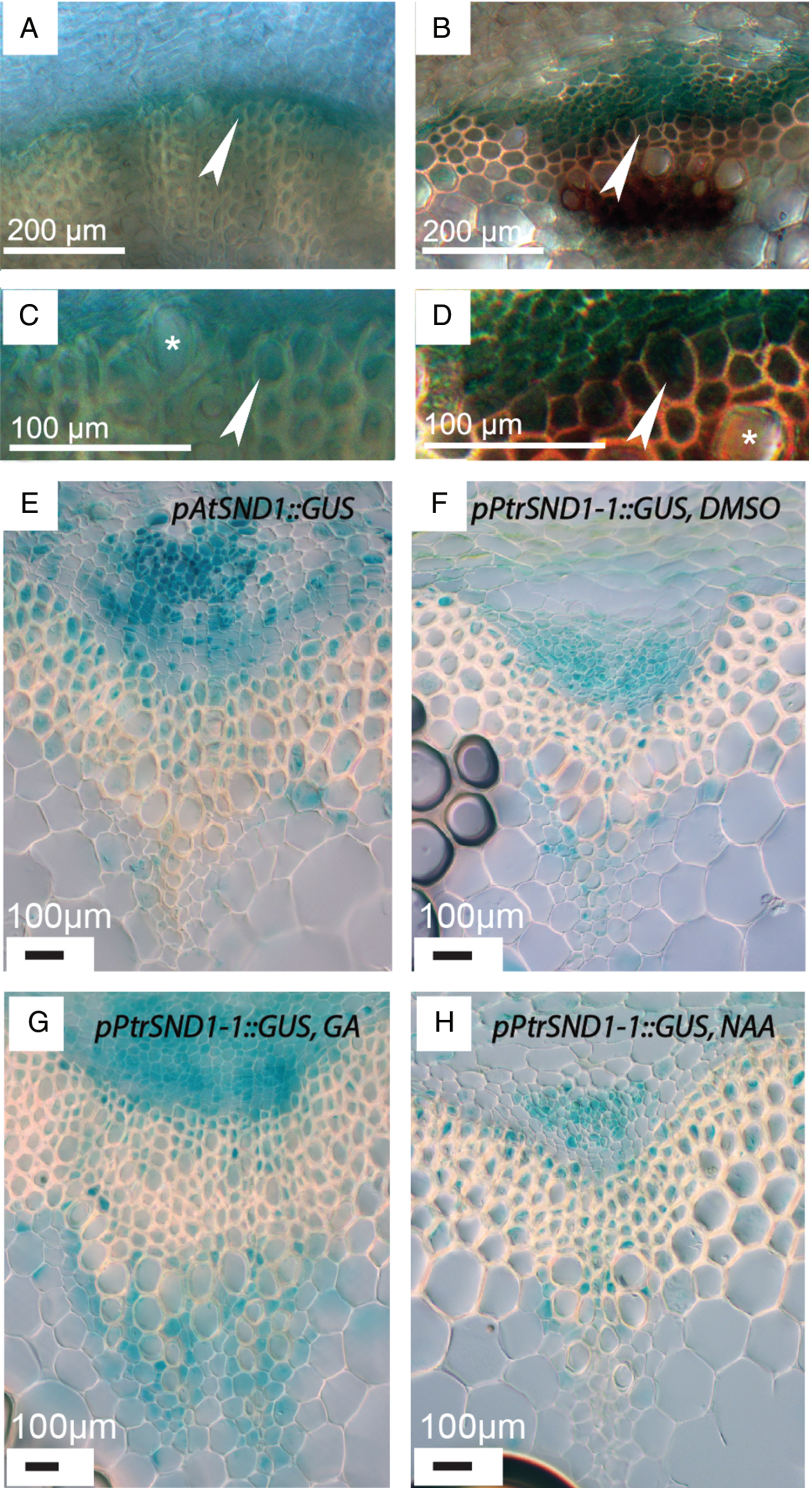
GUS signal from plants transformed with GUS under the control of a 315BP promoter fragment of *PttSND1‐1*. (A) and (C) GUS signal in hypocotyl of 30 days old *Arabidopsis thaliana*. Signal is visible in cells directly adjacent to, and inside fiber cells undergoing SCW deposition (arrow), but not in vessel cells (*). (B) and (D) GUS signal in inflorescence stems of 30‐day old *A. thaliana*. Signal is visible in cambial cells close to IF fibers (Arrow), as well as in adjacent fiber cells undergoing SCW deposition. No signal is visible in vessel cells. (E) Expression pattern of pAtSND1, untreated control. (F) Expression pattern of 315 bp pPtrSND1‐1 after 4 h DMSO treatment. (G) Expression pattern of 315BP pPtrSND1‐1 after 4 h GA treatment. (H) Expression pattern of 315 bp pPtrSND1‐1 after 4 h NAA treatment.

Together, the results of the in silico analysis pointed toward *PtrSND1‐1* encoding a transcription factor expressed during the early stages of fiber differentiation and SCW deposition in fibers. As it also had an overall high expression across wood sections as well as a low expression in the cambium, it became the focus of further study. To examine the expression pattern of this gene, we isolated a 1276 bp long promoter fragment of *PttSND1‐1* (*Potri.011G153300*) and employed it to drive expression of the GUS gene in *Arabidopsis*. We observed GUS signal in cambial cells adjacent to the xylem (Fig. S5) radially outward from vascular bundles in inflorescence stems, although no GUS signal was observed in the youngest, expanding and fully expanded vessels. To investigate the functional contributions of *cis*‐elements in proximity of the transcriptional start site, the promoter was truncated into a subfragment of 315 bp, from which we could observe more distinct GUS signal in the same regions, as well as in cells directly adjacent to the xylary cylinder in hypocotyls (Fig. 3A) and more weakly in the youngest fiber cells. We observed no GUS signal in the phloem or cortex of the hypocotyl. In inflorescence stems, the GUS signal was restricted to interfascicular (IF) fibers (Fig. 3B), as well as cambial cells directly neighboring vascular bundles. The expression pattern of *pAtSND1* (Fig. 3E) is centered on the cambial side of the cambium/xylem border, with GUS expression detectable in the outermost fiber cells; however, it declines rapidly in cells further in toward the pith. And as with the *PtrSND1‐1* promoter fragment, expression appears to be lacking in vessel cells. GUS signal is also detectable in a larger part of the cambium when driven by *pAtSND1* compared with *pPtrSND1‐1*.

An attempt at identifying *cis*‐elements using the PLACE database (Higo 1998) yielded a large number of hits, which were reduced to a list of eight (Appendix S1) by filtering expected random hits, irrelevant (e.g. root‐specific) motifs, and general transcription elements such as GATA‐box. Of particular interest were the presence of an auxin responsive element (ARFAT) and one GA‐responsive element (GAREAT; Table 1). Searching 1500 bp regions upstream of the transcriptional start site in SWNs, almost all promoters contain the elements found in the short promoter fragment, at roughly the same rate of incidence (Fig. S6). The GAREAT element is only present in one promoter of the *SND1*/*NST* homologs, while found in all investigated *VND*s, except in *PttVND6‐1* (*Potri.015G127400*).

**Table 1:** Distribution of filtered cis‐elements across an isolated 315 bp promoter fragment of *PttSND1‐1*.

Motif name	Strand	Start	Stop	Seq.	Reference
ARFAT	Pos	−153	−148	TGTCTC	Ulmasov et al. (1999)
ARR1AT	Pos	−265	−261	TGATT	Sakai et al. (2008)
GAREAT	Neg	−164	−158	TAACAAG	Ogawa (2003)
MYB1AT	Pos	−214	−209	AAACCA	Abe (2002)
MYCCONSENSUSAT	Pos	−226	−221	CACATG	Abe (2002)
MYCCONSENSUSAT	Neg	−226	−221	CATGTG	Abe (2002)
NAPINMOTIFBN	Pos	−54	−48	TACACAT	Ericson et al. (1991)
SORLREP3AT	Neg	−59	−51	TGTATATAT	Hudson and Quail (2003)

As a step to ascertain whether GA or NAA has an effect on the activity of this promoter, we subjected inflorescence stem pieces of *Arabidopsis* plants transformed with the 315BP *pPtrSND1‐1:GUS* construct to GA or NAA for 3.5 h. Comparing the expression patterns of *PtrSND1‐1* in *Arabidopsis* after short‐term treatment by GA3 or NAA revealed altered expression in comparison to the control treatment (DMSO; Fig. 3F, H). As mentioned, the pattern in DMSO‐treated *pPtrSND1‐1:GUS* is more restricted than in *pAtSND1:GUS* samples. Treatment with GA expands the *pPtrSND1‐1* expression pattern to one more similar to that of *pAtSND1* while addition of NAA further restricts the expression pattern compared with DMSO treatment.

**Figure 4:**
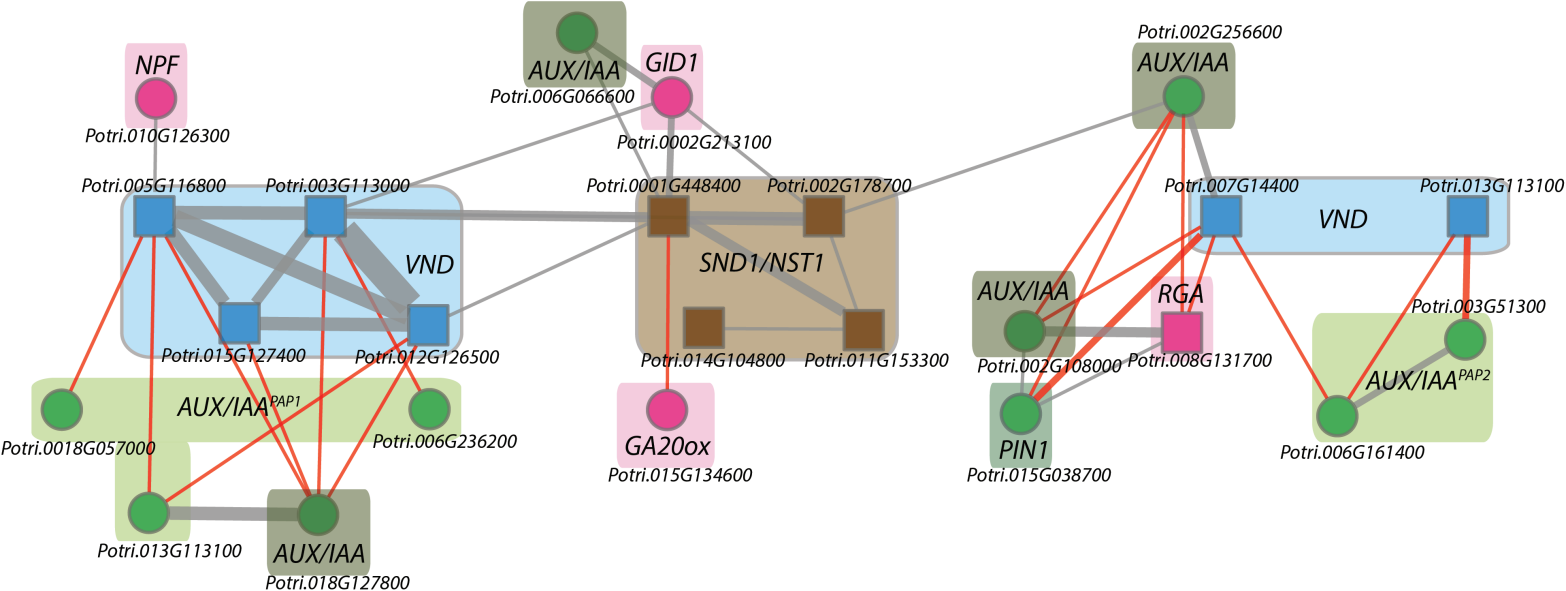
Co‐expression network involving *Populus* SWNs, *VND* homologous and auxin/GA‐related genes. Co‐expression network (threshold 4) illustrates the differential distribution of *Aux*/*IAA* (light green) genes across wood and their different correlation with *VND*s (blue) and *SND1*/*NST1* (brown)‐family genes. GA‐related genes (magenta) also differentially co‐vary across wood.

Given these results, we chose to examine whether the expression of auxin‐ or GA‐related genes co‐varies with the transcript levels of the *Populus* SWNs in developing wood. Querying AspWood for genes co‐expressed with at least one of the TFs in this set, we found a number of genes involved in the sensing, signaling and biosynthesis of these hormones (Fig. 4). *VND* homologous expressed close to the cambial zone, *PtrVND3‐2*, *PtrVND6‐1*, *PtrVND6‐2* and *PtrVND6‐4* (Fig. 4, left) co‐varied negatively with three homologs of the *Arabidopsis PHYTOCHROME-ASSOCIATED PROTEIN 1* (*PAP1*), one homolog of *INDOLE-3-ACETIC ACID INDUCIBLE 11* (*IAA11*), all of which are members of the *Aux*/*IAA* family of transcriptional repressors, and a homolog of the GA‐transporter *NITRATE TRANSPORTER/PEPTIDE TRANSPORTER 3* (*NPF3*), a member of the *NPF* family, which transports both active GA as well as its precursors and metabolites (David et al. 2016). The *SND1*/*NST1* homologs correlate negatively with a *GIBBERELLIN 20 OXIDASE 2* (*GA20OX2*), while the two *Populus SND1* genes both positively co‐vary with a homolog of *GA INSENSITIVE DWARF1b* (*GID1B*) and an *IAA11* homolog. For *PtrVND3‐1* and *PtrVND7‐1*, expressed further from the cambium, we observe additional *IAA11* homologs, as well as two *PAP2* genes, and a *REPRESSOR OF GA* (*RGA1*) gene, all but one negatively co‐varying with the two *VNDs*. Finally, a homolog of *PIN-FORMED 1* (*PIN1*) co‐varies with *VND3‐1* and also positively co‐varies with a *RGA1* gene.

**Figure 5:**
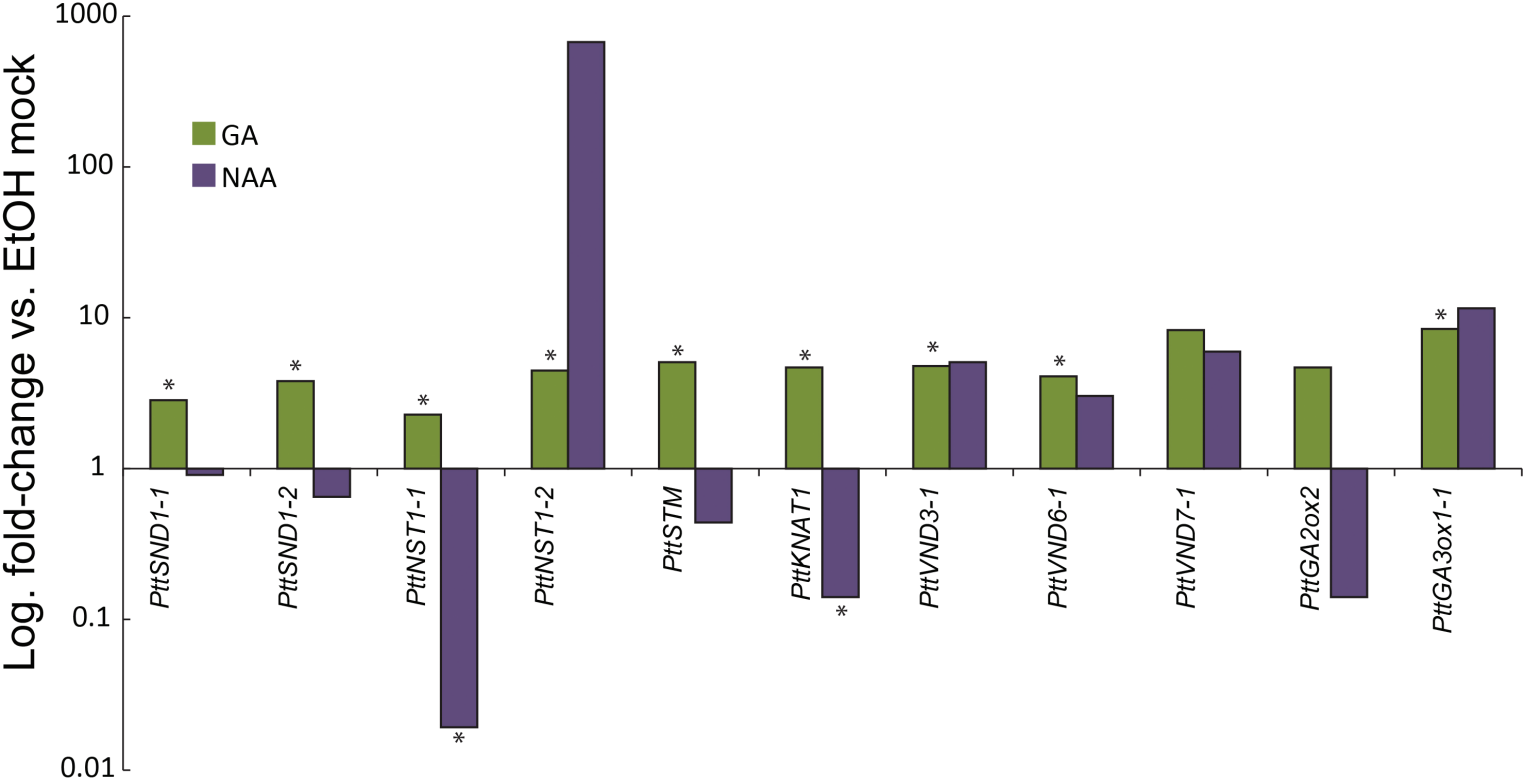
Quantification of transcriptional changes in response to GA or auxin treatment. GA treatment has a positive effect on the transcription of all tested genes, while auxin represses a *PttSND1‐1*, *PttSND1‐2*, *PttNST1‐1*, *PttSTM* and *PttKNAT1*. qRT‐PCR means of five independent replicates. Stars denote significant difference (*P* < 0.05, *t*‐test, n = 5). See Appendix S1, for gene accession numbers.

To test whether auxin and GA regulate expression of other SWN genes, we isolated and treated *Arabidopsis* stem pieces, as well as internodes of tissue‐cultured *Populus* cuttings undergoing secondary growth, for 3.5 h with either hormone. In *Populus*, the expression of *PttSND1*‐*1/2*, *PttNST1‐1/2*, *PttKNAT1*, *PttSTM*, *PttVND3‐1*, *PttVND6‐1* and *PttVND7‐1* was measured by qRT‐PCR. We noticed significant up‐regulation of all but *PttVND7‐1* after GA treatment (Fig. 5), although there is a tendency for the latter to also be induced. In line with previously published data (Björklund et al. 2007), a catabolic *GA2ox* was up‐regulated upon GA treatment (Fig. 5). In presence of auxin, the *SND1* orthologs were slightly repressed to between 0.9‐ and 0.65‐fold of the control, while the expression of *PttNST1‐1* was significantly repressed to 2% of the control level. In contrast, *PttNST1‐2* was strongly, but non‐significantly, induced upon auxin treatment. Similar to GA applications, expression of *PttVND* homologous was induced by auxin. As expected from previous findings in *Arabidopsis* (Hay and Tsiantis 2010), *class I KNOX* genes were down‐regulated in the presence of exogenous auxin.

To test whether the hormonal regulation of SWNs is conserved between *Populus* and *Arabidopsis*, we investigated expression of *NST1*, *SND1*, *SND2*, *VND3*, *VND6* and *VND7*, as well as *GA2ox2* and *GA3ox1*, in *Arabidopsis* inflorescence stems. We observe a similar trend in response of SWNs to GA as in *Populus* (Fig. S7), although the response to NAA differs between the two species (Fig. S7).

We proceeded to test whether GA or NAA treatment affects expression of the class I *KNOX* transcription factors *KNAT1* and *STM*, which are positive regulators of xylem fiber differentiation (Liebsch et al. 2014) and, in the case of *KNAT1*, has been shown to be regulated by auxin (Hay and Tsiantis 2010). In *Arabidopsis knat1* and *stm* mutants, expression of SWNs is strongly diminished (Liebsch et al. 2014). Both genes were induced by the GA treatment and repressed by NAA in a manner similar to that of the fiber‐specific *SND1* genes (Fig. 5).

**Figure 6:**
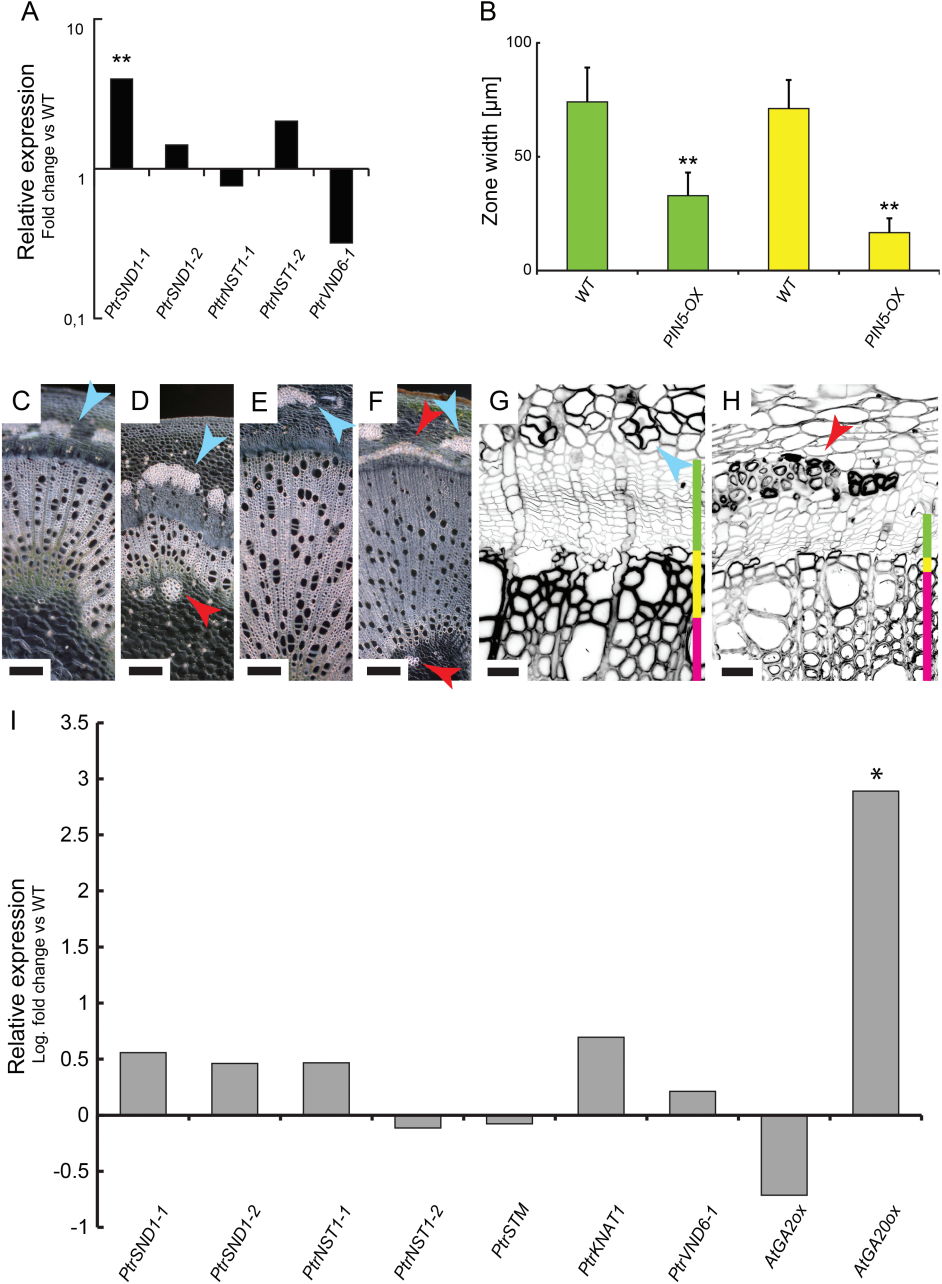
Anatomy and SWN gene expression in PIN5 and AtGA20ox‐expressing plants. (A) Expression of PtrSWNs in PIN5 OE relative to PIN1. (B) Width of developmental zones (μm) colored vertical bars denote developmental zones, Green = cambium, Yellow = non‐lignified cell wall zone. (C) Anatomy of Wt 10th internode. (D) Anatomy of PIN5 OE 10th internode. (E) Anatomy of Wt 20th internode. (F) Anatomy of PIN5 OE 20th internode. (G) False color, close‐up of calcofluor white stained 20th internode of Wt. (H) False color, close‐up of calcofluor white‐stained 20th internode of PIN5 OE. (I) Expression of PtrSWNs in *Populus* AtGA20ox OE. A, I. qRT‐PCR means of five independent replicates. Stars denote significant difference (*P* < 0.05, *t*‐test, n = 5). RNA extracted from the 10th internode.

Our findings prompted us to hypothesize that hormone‐induced changes in gene expression ought to be reflected in changes of cambial activity, cell differentiation, maturation and SCW deposition. In order to interfere with native auxin physiology, we overexpressed the auxin transporter *PtrPIN5* in *Populus*. *PIN5* localizes to the endoplasmic reticulum (ER) and transports auxin from the cytoplasm to the ER‐lumen (Mravec et al. 2009). Overexpression of *PIN5* is expected to retain auxin in source cells. Assuming same decay rate in transgenic and non‐transgenic plants, this may translate into lower auxin levels with increasing distance from the source. The most strongly overexpressing PIN5 line (Fig. S8B) was severely dwarfed. In the 20th internode of transgenic plants, an ectopic layer of phloem fibers was formed and the size of cambial‐elongation zone, and non‐lignified SCW zones decreased significantly (Fig. 6E, F). The latter finding is similar to the decapitation experiments. *PIN1* expression was significantly reduced as compared with wild‐type wood (Fig. S8). Relative to *PIN1* transcript levels *PtrSND1* was overexpressed, whereas relative *VND6* levels decreased in *PIN5OX* (Fig. 6A).

Disruption of endogenous GA availability was studied in *Populus* plants transformed with a *35S:AtGA20ox* construct. These plants produce more active GA, increased number of xylem fibers and more secondary growth (Eriksson et al. 2000, Mauriat and Moritz 2009). We found a general trend of increased expression of *PtrSWN*s (Fig. 6I) similar to GA‐treated decapitated plants. Exceptions to this trend were found in *PtrNST1‐2* and *PtrSTM*.

**Figure 7:**
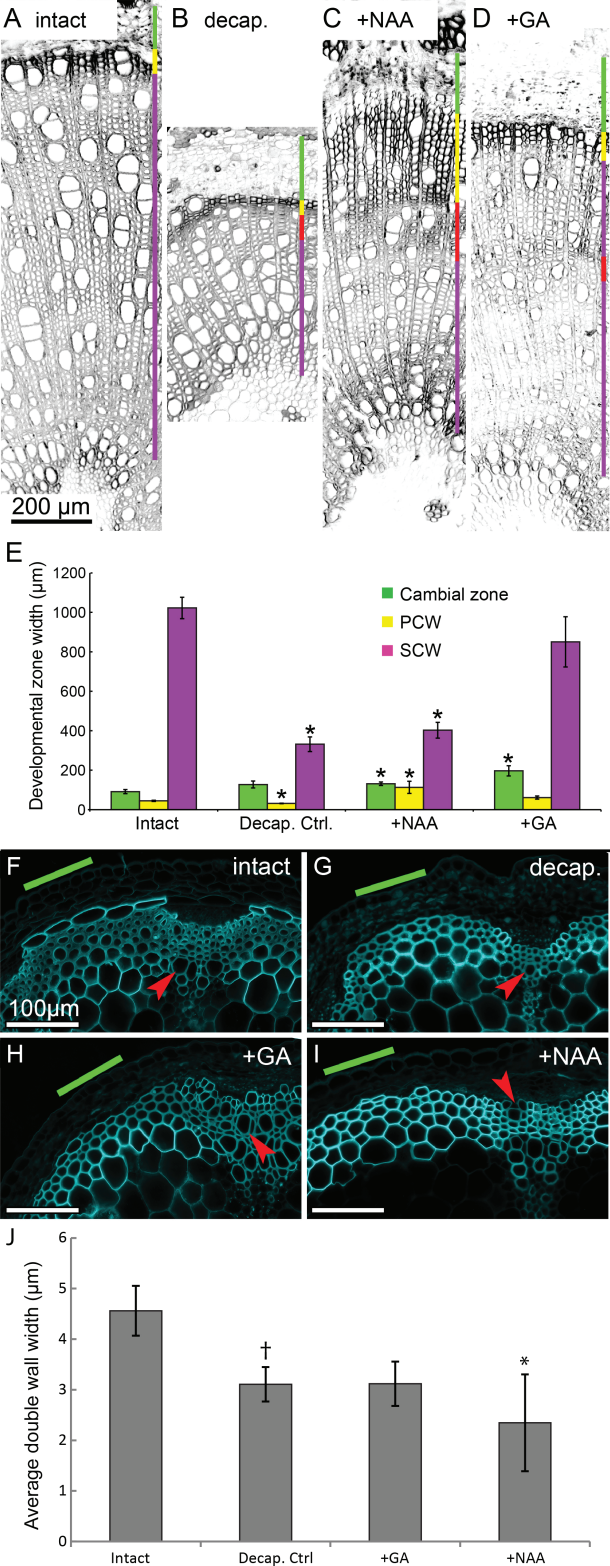
Effects of NAA or GA‐treatment on Populus and Arabidopsis anatomy. (A–D) Colored vertical bars denote developmental zones. Green = cambium, Yellow = non‐lignified cell wall zone, Purple = SCW deposition and cell maturation. Red bar denotes transition zone where temporary lack of apically supplied hormones due to decapitation has led to late wood‐like anatomy. (E) Widths of developmental zones under NAA or GA treatment. Changes in developmental zone width (μm) and the effect of NAA or GA treatment in Cambium (green), non‐lignified cell wall zone (yellow), SCW deposition/maturation zone (magenta). Stars denote significant difference vs zone width in intact controls (P > 0.05, n = 5), bars denote SE. (F–I) Calcofluor white‐stained transverse sections of Arabidopsis thaliana inflorescence stems. (F) Intact, (G) decapitated DMSO treated, (H) decapitated GA‐treated, (I) decapitated NAA treated. (J) Cell wall thickness in respective treatment. †Denotes significant difference compared with intact control. *Denotes significant difference compared with decapitated, DMSO‐treated control.

We then performed 3‐week treatments of decapitated *Populus* shoots and *Arabidopsis* inflorescence stems by either GA or auxin (NAA) dissolved in lanolin and applied to the site of decapitation. In *Populus*, decapitation resulted in rapid cessation of secondary growth (both cell division and expansion) of fiber and vessel cells, giving rise to a structure similar to latewood (Fig. 7B, red bar), with differentiated cells failing to fully expand but forming thick SCWs. Decapitation alone has little to no effect on shifting the ratio of cell identity toward either fibers or vessels (Fig. S9). Under both hormone treatments, there were distinct zones where we found smaller cells (Fig. 7C, D, red bars), as measured from center of cell lumen to the center of the neighboring cell lumen, which also have increased SCW thickness. When supplemented with NAA, the width of the cambial zone, i.e. the distance from differentiated phloem fibers to fully expanded xylem cells, the border of which we define as the first layer of cells displaying bright calcofluor signal (Fig. 7C, green bar) was partially rescued, implying an increase in cambial cell division and/or cell expansion rate. We also observed a restoration of cell expansion of both xylem vessels and fibers upon NAA treatment compared with decapitated controls. Meanwhile, cell wall lignification and thickening appears to be impaired in cells formed radially outside of the TZ (Fig. 7C, yellow bar). After NAA treatment, we observed a shift in vessel to fiber (V/F) ratio (Fig. S9). However, the total number of observed vessels did not shift.

In GA‐treated stems, we observed a zone of growth directly radially outside of the TZ, in which vessel formation is impaired only to be restored toward the outermost part of the xylem, corresponding to the end of treatment. Comparison of V/F ratio before and after treatment revealed a statistically significant shift as there was a decrease in the number of vessel cells per fiber after treatment with GA (Fig. S9). There is also a statistically significant decrease in the total number of vessel cells per unit area observed compared with the mock‐treated control.

As in the NAA‐treated plants, the width of the cambial zone is partially restored after GA treatment compared with decapitated controls and is indeed at times greater than the cambium measured in intact controls (Fig. 7D, green bar). The width of the 10 fibers closest to the cambial zone is also significantly greater than in decapitated controls, and indistinguishable from intact controls, indicating that both cambial cell division rate and cell expansion are partially restored upon treatment with GA.

The transition from non‐lignified to mature, lignified, SCW is gradual in the intact control plants and while GA‐treated plants display anatomy similar to that of intact controls (Fig. 7D), we observe a wider non‐lignified zone in NAA‐treated samples (Fig. 7, yellow bar), which has a sharp boundary with the mature zone, often at, or close to, the TZ.

Decapitation of *Arabidopsis* resulted in disruption of development similar to what was observed in *Populus*, although mainly characterized by a lack of cell wall maturation (Fig. 7G) as evidenced by thinner SCWs, displaying brighter calcofluor white signal than in non‐decapitated controls (compare Fig. 7F, G). Decapitated plants also consistently failed to initiate development of the starch sheath into cambial cells, which was ongoing in samples of intact plants (Fig. 7G). Supplementation of decapitated plants by either GA or NAA does not fully rescue these phenotypes; however, SCWs in plants supplemented with GA are significantly thicker and display a weaker calcofluor white signal than those in plants supplemented with NAA (Fig. 7H–J), in line with the observations in *Populus*.

## Discussion

We reason that using the tools developed and described by Sundell et al. (2017) as well as data presented in the same, we are able to accurately select candidate SWN involved in distinct process of wood formation, avoiding false positive selection of phylogenetically closely related genes, whose functions may have diverged from those established in *Arabidopsis*, as a result of neo‐functionalization subsequent to the Salicaceae WGD.

The clear distinction between genes of the *VND*, *SND1*/*NST1* and *SND2*/*3*‐clades evident in the phylogenetic tree of Fig. 1 is, on the whole, reflected on the expression level. If we consider cell distance from cambium as a proxy for cell age, it is clear that the *VND6*‐homologous are induced in younger cells, i.e. recently divided cambial xylem initials, while *SND1*/*NST1* homologous are expressed in slightly older cells, and *SND2*/*3* homologous, targets of *SDN1*/*NST1*, are expressed in still older cells. The distinct clustering of *PtrSND1*, *PtrSND2* and *PtrNST1* homologous together with SCW‐associated *CESA*s (Fig. 2), *PtrVND6* and *PtrVND3‐1* with PCW *CESAs* and other cell wall component biosynthetic genes, and *PtrSND3* homologous negatively correlating with PCW biosynthetic genes further illustrates the hierarchical nature of the control of xylem cell wall deposition and how this manifests in space and time. And as induction of SCW biosynthesis‐related gene *CESA7* expression coincides with that of *SND2*/*3*, indicating a closer transcriptional relationship with these genes than to *SND1*/*NST1*, suggesting that the *SND1*/*NST1* homologs play a role in the specification or early development of fibers rather than in the direct regulation of SCW deposition as suggested previously (Sundell et al. 2017). The latter regulatory function is more likely performed by *SND2* homologous, as indicated by their tight association with SCW biosynthetic genes.

The fact that expression profiles within paralogous pairs are similar indicates that there has been no drastic neo‐functionalization, with the exception of the *Populus VND7* homologous. *PttVND7‐1* (*Potri.013G113100*) is sharply induced in the zone of cell maturation/PCD while the second ortholog (*Potri.019G083600*) is not found to be expressed at all in secondary xylem, although investigation of the transcription throughout the plant (Fig. S4) reveals that it is expressed in tissues with primary vasculature. This may hint at that in *Populus*, unlike in *Arabidopsis*, PCD of tracheary elements and cell‐type specification is initiated by differentiated *VND6* and *VND7* genes. All this allowed us to confidently select a candidate, *PtrSND1‐1*, for further study of the control of SCW formation in xylem fibers.

Study of isolated promoter fragments from *PtrSND1‐1* driving GUS expression in *Arabidopsis* confirms that this promoter is specifically active in fibers of both the hypocotyl and inflorescence stem, and in silico evidence points to its function being modulated by the hormones auxin and GA. Building on previous studies (Immanen et al. 2016), we used the AspWood tool to investigate whether or not proteins involved in auxin or GA biosynthesis, or signal mediation co‐vary with the NAC domain TFs involved in xylem development. Also, we found that different *Aux/IAA* genes co‐vary with the *VND*‐family genes across wood (Fig. 4), while the GA receptor *GID1B* positively co‐varies with *PtrSND1‐2*. The distinct expression domains of the different *Aux*/*IAA* genes and their specific interaction with different classes of *SWN*s may indicate that the response to auxin varies across wood zones. In such a model, different combinations of *Aux/IAAs* and *TIR1/AFBs* with different binding affinities to auxin would attribute to a differentiation zone‐specific auxin response. Scaled auxin sensitivity in different parts of wood would require the expression of relevant receptors to be under the control of a signal other than auxin itself.

In PIN5 OE plants, where auxin is likely retained longer in cells in which it is produced, rather than entering the polar auxin transport chain, we observed stunted growth, increased relative expression of *PtrSND1‐1* and reduced levels of *PtrVND6‐1*. The latter could affect vessel differentiation negatively and as a consequence result in stunted growth. In line with the transcriptional changes in PIN5 OE, treatment of *Populus* internodes with auxin leads to suppressed expression of regulators of fiber differentiation, while there is a trend for determinants of vessel differentiation to be induced by the same hormone. GA, on the other hand, appears to act as a general inducer of expression of tested SWN genes, a function that is in line with its role in cellular expansion and maturation. Effect of NAA and GA treatment on expression of *STM* and *KNAT1* implies that these TFs form part of the regulatory mechanisms of fiber differentiation and subsequent SCW deposition. This is in line with the observation that the *knat1* mutant is partially resistant to GA and that *KNAT1* is required for expression of *SND1*/*NST1* genes (Liebsch et al. 2014, Ikematsu et al. 2017).

The differences in gene expression on treatment by GA or NAA are reflected in anatomy of *Populus* stems. In decapitation experiments, loss of the apical hormone source initially leads to the formation of late wood‐like structures, which we identify as transition zones, characterized by consecutively smaller cells with thick cell walls, which is later partially rescued as supplemented hormones supposedly reach the tissue. This allows to identify the start of treatment and to compare between before and after states of, e.g. tissue composition. Staining of samples with calcofluor white, allows quantification of differences between non‐lignified and lignified SCW zones, as the binding of the fluorophore may be affected by lignin and a brighter signal originates in the non‐lignified zone (Pradhan Mitra and Loque 2014).

Our experiments indicate that while NAA is able to drive cambial cell division and does not seem to impair early fiber or vessel differentiation, it is not sufficient for proper initiation of the SCW biosynthetic program as SCWs are both thinner and appear less lignified in the post‐decapitated samples, than in ones supplemented with GA. Nor does NAA fully rescue cell expansion, leading us to conclude that the apparent shift in V/F ratio may be a result of a greater number of smaller fiber cells per unit area skewing the calculation. Conversely, supplementation with GA does rescue cell division, expansion and secondary wall deposition, although plants treated with GA initially form significantly fewer vessels compared with mock‐treated plants, leading us to conclude that fewer cambial cells differentiate into vessels on removal of the apical auxin source and that GA supplementation is insufficient to trigger normal vessel differentiation in *Populus*.

The long‐term hormone supplementation experiments in both *Populus* and *Arabidopsis* illustrate that the SCW regulatory mechanism may be conserved between the two species, although *Populus* likely has adapted a more fine‐tuned version, utilizing divergent SWNs for various functions. What is clear from our experiments is that NAA‐treatment appears to drastically reduce the thickness and lignification of the SCW in both species. While GA‐treatment is not able to fully rescue wild‐type phenotype (compare Fig. 7F, H) cells appear more expanded than in decapitated controls (Fig. 7G), indicating at least a partial rescue of growth. Interestingly, SCWs of vessel cells in decapitated and hormone supplemented *Arabidopsis* appear to differentiate normally, irrespective of treatment (Fig. 7F–I, red arrows).

Taken together with the gene expression results for *PtrSND1*s and *PtrNST1*s, our data fortify the case of auxin as a negative regulator of SCW formation in trees, possibly acting through repression of NAC‐domain transcription factors specifying fiber identity and/or directly regulating SCW biosynthesis. Our data suggest that a high concentration of auxin in the cambial and expansion zones represses fiber‐specific SWNs and permits the differentiation of xylem vessels, followed by a general induction of all SWNs and fiber specification once auxin is depleted and active GA levels are high. The underlying mechanisms of how auxin and GA co‐ordinate SWN expression remain unclear. Interestingly, GA and auxin response elements appear to overlap in the *PtrSND1‐1* promoter. This promoter architecture could provide a simple mechanism, for the suggested GA‐auxin crosstalk, driven by competitive binding of GA‐ and auxin‐response activators and repressors, respectively. Novel genome‐editing techniques may allow for future studies of this hypothesis in *Populus* by selective mutation of these *cis*‐elements. This, in turn, could yield interesting potential applications for regulating gene expression specifically in fiber cells or for alleviating problems encountered when attempting to engineer wood quality where vessel integrity is compromised.

## Author contributions

C.J. performed the experimental work for the manuscript except for the experiments involving *35S::PIN5* (X.J. and C.D.) and *AtSND1::GUS* (W.X. and L.L.). C.J. and U.F. designed the experiments and interpreted collected data. C.J. wrote the manuscript supervised by U.F.

## Supporting information


**Appendix S1.** Accession numbers.

**Fig. S1.** Heatmap of all wood‐expressed *PtrNAC*
**Fig. S2.** Venn diagram of overlapping, directly co‐regulating neighbors to PtrSND1‐1, PtrSND1‐2, PtrSND2‐2, PtrVND6‐3 and PtrVND7‐1
**Fig. S3.** Promoter fragments distribution and length compared to *PtrSND1-1*
**Fig. S4.** Expression profiles PtrVND7‐1 and PtrVND7‐2
**Fig. S5.** GUS Expression long fragment in *Arabidopsis thaliana*
**Fig. S6.** *cis*‐element distribution in 1.5 kb promoters
**Fig. S7.** Log fold‐change in expression of *AtSWN*s on treatment with either GA or NAA
**Fig. S8.** Phenotype, PIN5 expression measurement, in PIN5 OE lines
**Fig. S9.** V‐F Ratio in GA or NAA treated *Populus* internodes
