# Supplementary material for: The plant hormone auxin directs timing of xylem development by inhibition of secondary cell wall deposition through repression of secondary wall NAC‐domain transcription factors
Source: Physiol Plant. 2018 Aug 2;165(4):673–89. doi: 10.1111/ppl.12766 (PMC7379297; doi:10.1111/ppl.12766)
Supplement: Supplementary file 2 — Fig. S1. Heatmap of all wood‐expressed PtrNAC Fig. S2. Venn diagram of overlapping, directly co‐regulating neighbors to PtrSND1‐1, PtrSND1‐2, PtrSND2‐2, PtrVND6‐3 and PtrVND7‐1 Fig. S3. Promoter fragments distribution and length compared to PtrSND1-1 Fig. S4. Expression profiles PtrVND7‐1 and PtrVND7‐2 Fig. S5. GUS Expression long fragment in Arabidopsis thaliana Fig. S6. cis‐element distribution in 1.5 kb promoters Fig. S7. Log fold‐change in expression of AtSWNs on treatment with either GA or NAA Fig. S8. Phenotype, PIN5 expression measurement, in PIN5 OE lines Fig. S9. V‐F Ratio in GA or NAA treated Populus internodes [file PPL-165-673-s002.pdf]

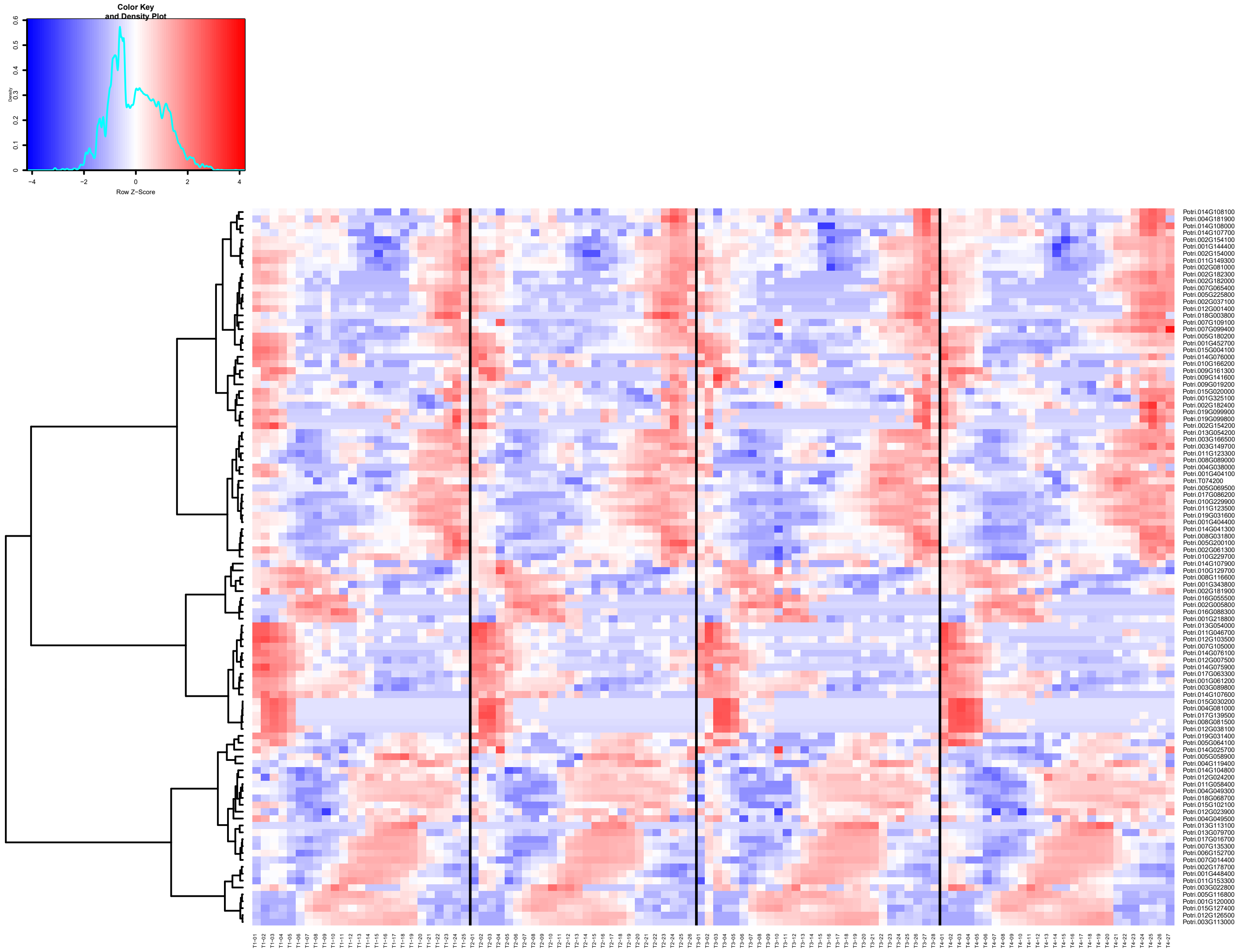

Supplemental Figure 1. Expression (VST) of Populus NAC genes across wood.

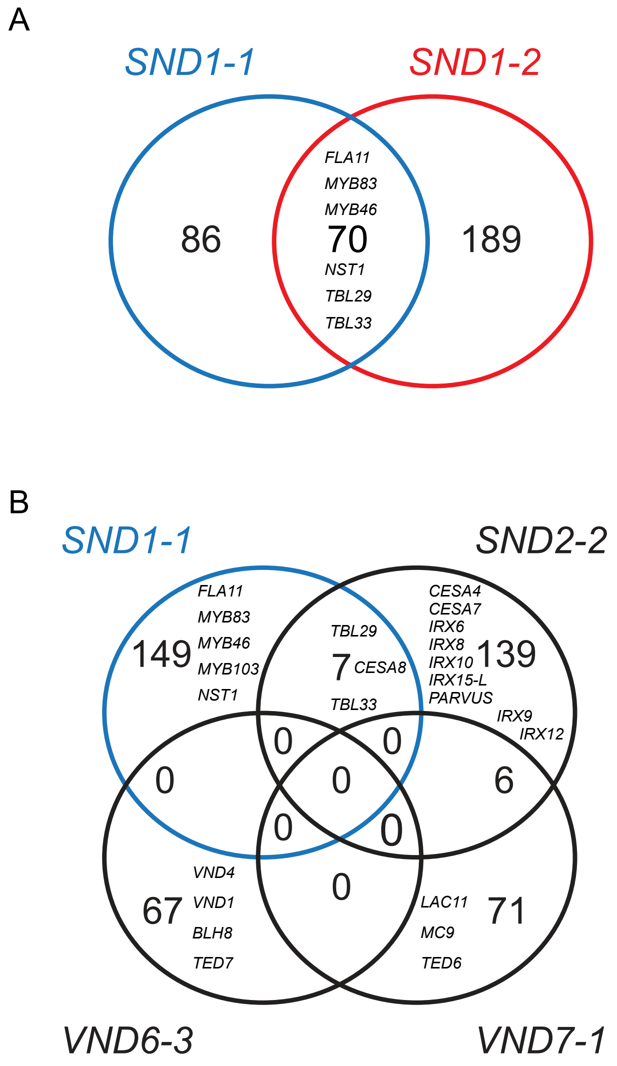

Supplemental Figure 2. Overlap of genes co-regulated with A, *SND1-1* and *SND1-2*; B, *SND1-1*, *SND2-2*, *VND6-3* and *VND7-1*-

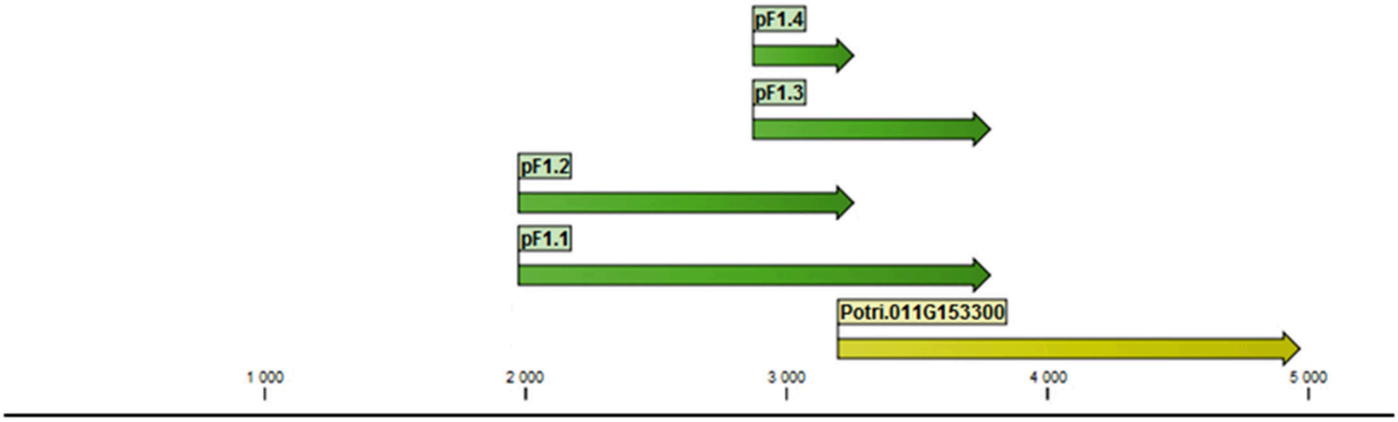

Supplemental Figure 3. Promoter fragment (green) distribution and length compared to *PtrSND1-1* (Potri.011G153300, yellow).

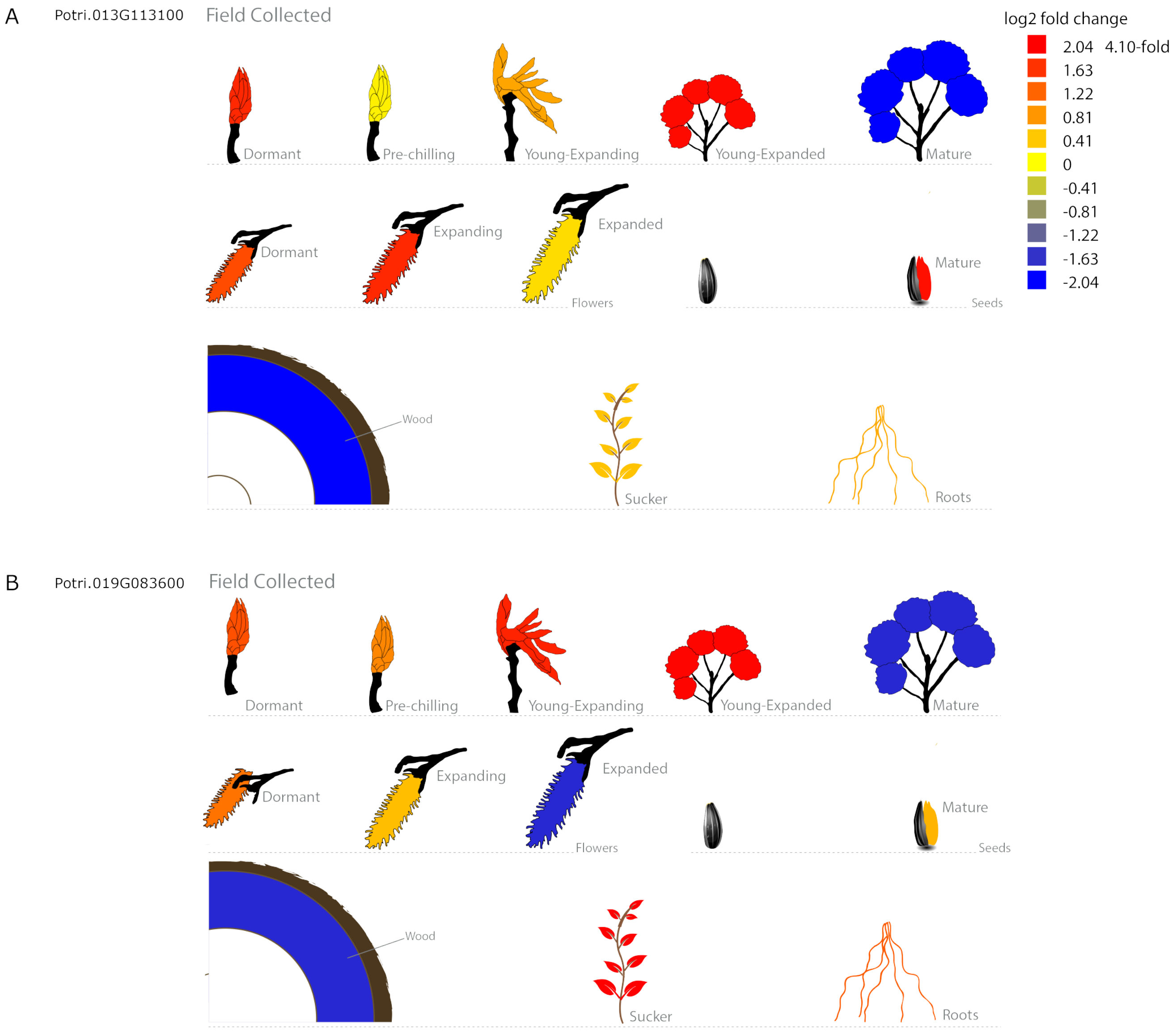

Supplemental Figure 4. Expression domains of Populus VND7-1 (A) and VND7-2 (B).

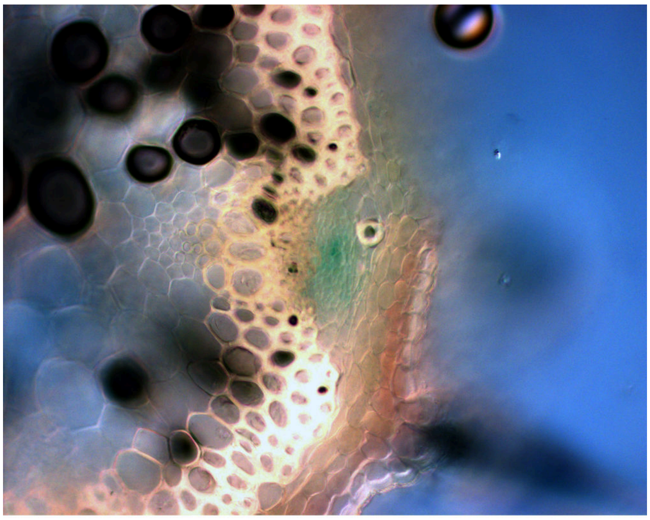

Supplemental Figure 5. GUS signal in an Arabidopsis inflorescence stem. Plant transformed with long 1276 bp promoter fragment from *PtrSND1-1* driving expression of *GUS*.

Name	Motif	AtSND1	AtNST1	PtrSND1-1	PtrSND1-2	PtrNST1-1	PtrNST1-2	AtVND7	AtVND6	PtrVND1	PtrVND3	PtrVND4	PtrVND5	PtrVND6
ARFAT	TGTCTC	1	1	1	1	1	0	0	2	0	1	0	2	0
ARR	*GATT	14	15	8	8	16	8	15	17	24	18	14	9	16
GAREAT	TAACAAR	0	0	1	0	0	0	1	2	1	1	1	1	0
MYBre	WACCA	2	1	2	3	2	1	2	4	2	4	4	1	1
MYCre	CA**TG	10	18	18	10	14	6	12	6	4	6	10	6	14
NAPINmotif	TACACAT	2	1	1	0	0	0	0	2	0	0	0	0	0
RAV1at	CAACA	3	2	1	3	3	1	7	4	3	3	4	0	1
SORLREP3	TGTATATA	2	0	1	0	0	0	3	0	0	0	0	2	1
SURECORE	GAGAC	1	1	5	2	2	2	0	3	1	1	0	3	0

Supplemental Figure 6. CIS-element incidence and distribution in 1.5 kb promoter fragments of Populus and Arabidopsis wood NAC TFs.

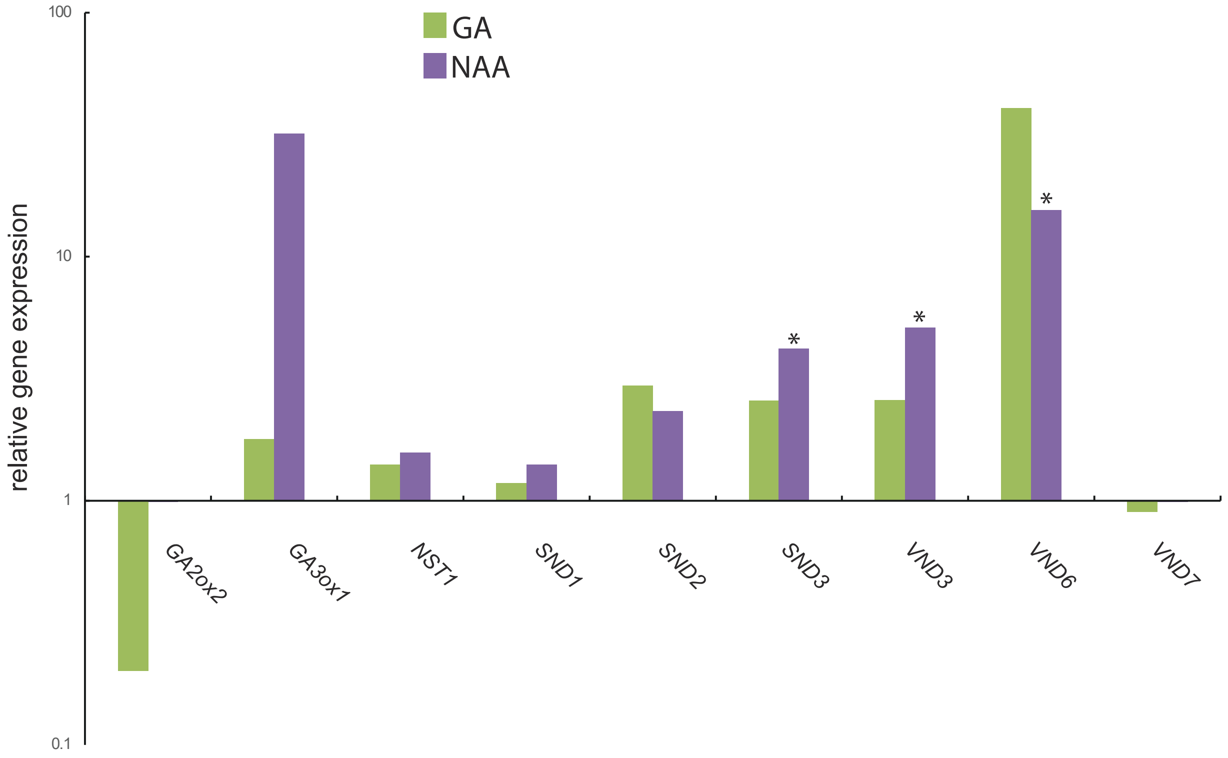

Supplemental Figure 7. Log fold change in expression of AtSWNs on treatment with either GA or NAA. Stars denote statistically significant difference vs. mock treatment (t-test, $p < 0.05$, $n = 5$).

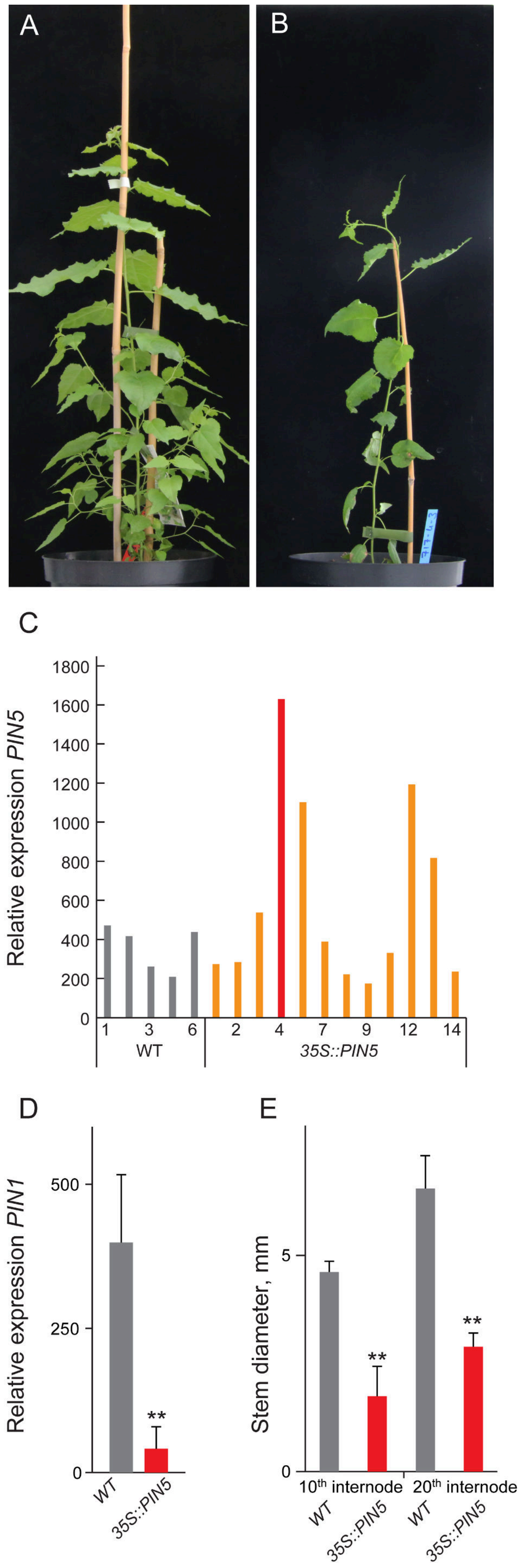

Supplemental Figure 8. Phenotype of WT plant (A), and *PIN5 OE*, line 4 (B). C, relative expression *PIN5* in *PIN5 OE* lines compared to WT lines. D, relative expression of *PIN1* in *PIN5 OE* line 4. D. Stem diameter of WT vs. *PIN1 OE* line 4, at the 10th and 20th internode.

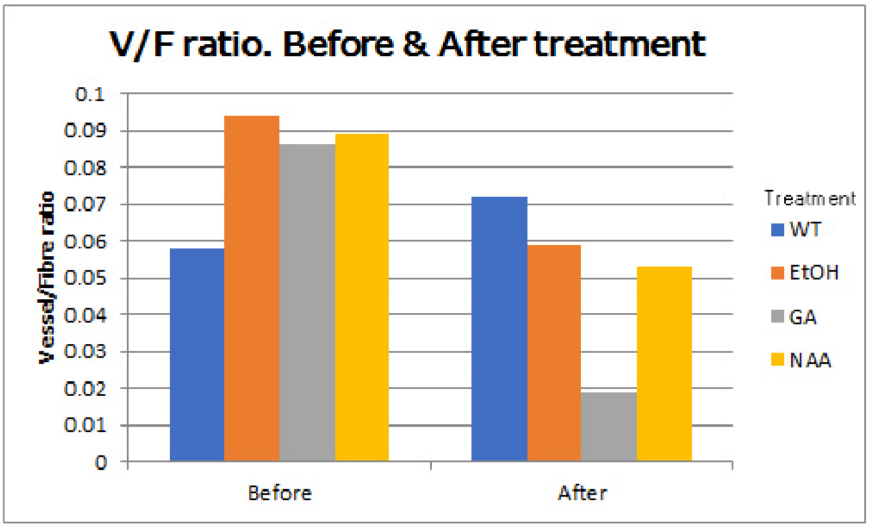

Supplemental Figure 9. Vessel to fibre ratio scored in equal areas of pre- and post-treatment sections of Populus wood.
